# The Improvement of the Performance of Sky-Blue OLEDs by Decreasing Interface Traps and Balancing Carriers with PSVA Treatment

**DOI:** 10.3390/polym14030622

**Published:** 2022-02-05

**Authors:** Yijun Ning, Suling Zhao, Dandan Song, Bo Qiao, Zheng Xu, Yuxiang Zhou, Junfei Chen, Wageh Swelm, Ahmed Al-Ghamdi

**Affiliations:** 1 Key Laboratory of Luminescence and Optical Information, Beijing Jiaotong University, Ministry of Education, Bejjing 100044, China; 18118039@bjtu.edu.cn (Y.N.); ddsong@bjtu.edu.cn (D.S.); boqiao@bjtu.edu.cn (B.Q.); zhengxu@bjtu.edu.cn (Z.X.); 19121666@bjtu.edu.cn (Y.Z.); 17118458@bjtu.edu.cn (J.C.); 2 Department of Physics, Faculty of Science, King Abdulaziz University, Jeddah 21589, Saudi Arabia; wageh1@yahoo.com (W.S.); AGAMDI@kau.edu.sa (A.A.-G.)

**Keywords:** PSVA, solution-processed, small-molecules, blue organic light-emitting diodes, thermally activated delayed fluorescence

## Abstract

The mitigation of interfacial charge accumulation in solution-processed organic light-emitting diodes (s-OLEDs) is an effective method to improve device performance. In this study, the polar solvent vapor annealing (PSVA) method was used to treat two layers in s-OLED, PEDOT:PSS and mCP:DMAC-DPS emitting layers, separately, to optimize the carrier transmission and balance. After the double-layer PSVA treatment, the current efficiency increased, the lifetime of the device is improved, the efficiency roll-off alleviated from 33.3% to 26.6%, and the maximum brightness increased by 31.3%. It is worth mentioning that the work function of the EML interface reduced by 0.36 eV, and the initial injection voltage of the electrons also reduced. Simulating the solubility of the LUMO and HOMO molecule parts of the mCP and DMAC-DPS, it was found that the LUMO parts had stronger polarity and higher solubility in polar solution than the HOMO parts. By comparing the untreated luminescent layer films, it was found that the PSVA treatment improved the uniformity of the film morphology. We may infer that a more ordered molecular arrangement enhances carrier transport as the LUMO parts tend to be close to the surface and the reduced local state traps on the EML surface promote electron injection. According to the experimental results, the injection of holes and electrons is enhanced from both sides of the EML, respectively, and the charge accumulated at the interface of s-OLEDs is significantly reduced due to the improvement of carrier-transported characteristics.

## 1. Introduction

Organic light-emitting diodes (OLEDs) based on thermally activated delayed fluorescence (TADF) emitters have great commercial potential in flat panel displays and solid lighting applications. These OLED products are mainly produced by the traditional vacuum-evaporation process, in which the lower quality and yield of products and the high cost of manufacture restrict the further development of OLED industrialization. Comparatively, the solution method is a competitive and attractive technology due to its clear advantages of large-scale manufacturing, low cost and flexible control of the doping ratio [1]. However, solution-processed OLEDs need to remove used solvents before forming films. In this process, phase separation and molecular aggregation occur due to molecular movement [2,3]. In addition, considering the inter-layer miscibility, it is unrealistic to add multi-functional layers to guarantee optimized layering, which will lead to many serious interface problems and make the situation worse. The molecular arrangement of organic material films, especially TADF small-molecule material films, is a key factor affecting the carrier transport of the whole device [4,5,6]. Compared with phosphorescent materials, TADF materials do not require heavy-metal atoms, and adjust their energy gap (ΔE_ST_) between singlet excitons (S1) and triplet excitons (T1) to produce an efficient reverse intersystem crossing (RISC) process. Therefore, triplet excitons can be upconverted to singlets by RISC in TADF materials with a small ΔE_ST_, which then emit fluorescence through radiation transition, so that in theory, the internal quantum efficiency (IQE) can reach 100%. However, due to the molecular aggregation of TADF OLEDs by solution processing and the long triplet exciton lifetime of TADF materials, the quenching problem of triplet–triplet annihilation (TTA) and triplet-polaron quenching (TPQ) in devices is acute, which seriously reduces the efficiency of TADF OLEDs at high current density [7,8]. In order to improve the efficiency of the roll-off of TADF devices, two essential problems need to be solved. One is to reduce the density of stored charge in the device by improving the carrier transport, and the other is to speed up the extinction of triplet excitons by accelerating the RISC rate. In recent years, scientists have devoted themselves to developing new materials and producing smaller ΔE_ST_ to accelerate the RISC rate [9,10]. However, the efficiency roll-off caused by the imbalance between two carriers cannot be ignored, especially in solution-processed devices. [11] Considering that spin coating may wash away or dissolve the upper films, solution-processed OLEDs need to simplify the spin-coating steps, and at the same time promote carrier injection and optimize charge balance. The morphology of the organic-layer film is the decisive factor in charge transfer [12]. In the process of organic films changing from liquid phase to solid phase, molecules move under the influence of the electrostatic field force, glass-transition temperature, solubility, viscosity, etc. Similar molecules prefer to aggregate together in a mixed solution to form a film, which results in phase separation and disordered molecular arrangements in the film [13]. The rough film morphology, disordered molecular distribution and separated phase of EML can hinder the carrier transmission, cause charges accumulated at interfaces of s-OLEDs, and increase the non-radiative relaxation of excitons.

At present, thermal annealing is the most commonly used method for preparing solution-processed OLEDs, which can effectively reduce the roughness of the film [14], but the molecular arrangement direction cannot be improved. Besides thermal annealing, polar solvent vapor annealing (PSVA) is also a potential post-treatment method [15,16,17,18,19,20]. Yeo et al. found that the PSVA method can reduce the injection barrier of the PEDOT:PSS layer into EML [17]. Cun et al. considered that PSVA forms a dipole layer on the surface of polymer emitter film [21]. In contrast to polymers, small organic molecules do not have long chain traction, but groups of small molecules also have differences in polarity. We speculated that the polarity difference in molecular groups would be reflected in the movement of molecules during PSVA, thus promoting the directional and orderly arrangement of different molecules. In this study, we treated PEDOT:PSS and mCP: DMAC-DPS films by combining thermal annealing with polar-solvent vapor annealing. Without adding other functional layers, the device based on ITO/PEDOT:PSS/mCP: DMAC-DPS/DPEPO/TPBi/LiF/Al improves the injection and transfer of holes and electrons respectively. Because of the balance of the two carriers, the current efficiency and the efficiency roll-off are also improved.

## 2. Materials and Methods

The guest material 10,10′-(4,4′-Sulfonylbis(4,1-phenylene))bis(9,9-dimethyl-9,10-dihydroacridine) (DMAC-DPS) was purchased from Taiwan Luminescence Technology Corp in Taiwan. The hole injection material, poly(3,4-ethylenedioxythiophene): poly (styrene sulfonate) (PEDOT:PSS), host material 1,3-Di-9-carbazolylbenzene Synonym (mCP), exciton blocking layer material Bis(2-(diphenylphosphino)phenyl)ether oxide (DPEPO), electron transport materials 1,3,5-Tris(1-phenyl-1H-benzimidazol-2-yl)benzene (TPBi), were purchased from Xi’an Polymer Light Technology Corp in Xi’an, China. All the above materials were used without further purification. mCP is widely used as a bipolar host material for EML of bule-TADF OLEDs for its high triplet exciton (T1) energy of 2.9 eV.

The indium tin oxide (ITO) substrates were cleaned in ultra-sonification with acetone, absolute ethyl, and deionized water in turn, then surface-treated with plasma for 90 s. The PEDOT:PSS was spin-coated at 4000 revolutions per minute (rpm) on ITO substrates in air and annealed at 150 °C for 15 min; next, the other layers were all created in the glove box with a nitrogen atmosphere. The TADF material DMAC-DPS, as the dopant, and mCP, as the host, were dissolved in chlorobenzene (CB) at a concentration of 10 mg/mL and mixed together at a ratio of 20 wt%. The EML mixed solution was spin-coated on PEDOT:PSS at 2000 rpm for 45 s and annealed at 60 °C for 25 min. Finally, the films of DPEPO, TPBi, LiF, and Al were prepared by thermal evaporation under a vacuum of 5 × 10^−4^ Pa and an evaporation rate of about 0.5 Å/s.

For the post-processing of solution-processed films, we used the polar-solvent vapor annealing (PSVA) method on some samples. We chose methanol as the polar solvent in the experiment because of its strong polarity and volatility. To make sure the selected sample was exposed to saturated methanol vapor for annealing, a large Petri dish was buckled upside down on a small Petri dish filled with solvent. After the large Petri dish was placed on the heating platform, liquid drops appeared on it. The sample to be post-treated was placed on the large Petri dish. PEDOT:PSS layer and EML are respectively annealed in the fume hood and the glove box filled with nitrogen. In order to eliminate the influence of the temperature of the atmosphere on the experiment during annealing, the contrast device that only needed thermal annealing was also covered with large petri dishes.

## 3. Results and Discussion

The details of the multilayer thin film stacks of the optimized general structure as the contrast device are as follows: 1: ITO/PEDOT:PSS/mCP: 20 wt% DMAC-DPS/DPEPO (10 nm)/TPBi (30 nm)/LiF (1 nm)/Al (100 nm).

In order to analyze the influence of PSVA on PEDOT:PSS and EML more clearly, the following three devices were produced for comparison. The use of M in brackets indicates that the annealing of this film was in methanol steam.

Device 2:

ITO/PEDOT:PSS/mCP: 20 wt% DMAC-DPS(M)/DPEPO (10 nm)/TPBi (30 nm)/LiF/Al

Device 3:

ITO/PEDOT:PSS(M)/mCP: 20 wt% DMAC-DPS/DPEPO (10 nm)/TPBi (30 nm)/LiF/Al

Device 4:

ITO/PEDOT:PSS(M)/mCP: 20 wt% DMAC-DPS(M)/DPEPO (10 nm)/TPBi (30 nm)/LiF/Al

**Figure 1:**
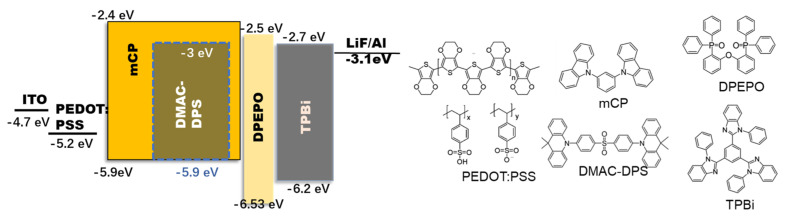
Schematic diagram of energy level structure of OLEDs and the chemical structure of materials.

The schematic energy level diagram of the fabricated device structure is shown in Figure 1.

**Figure 2:**
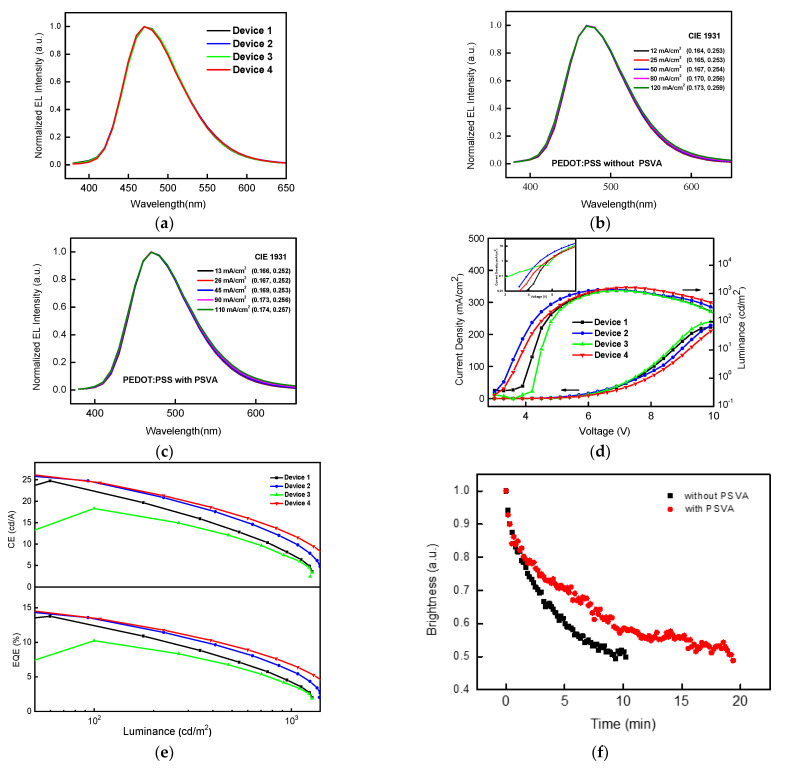
(**a**) The normalized EL spectra of devices at the same brightness, EL spectra and CIE coordinates of device 1 (**b**) and device 4 (**c**) with the variation of applied current densities, (**d**) J-V-L characteristics (the illustration in the figure shows I-V curve of logarithmic coordinates of low voltage), (**e**) CE-L, EQE-L curve of the as-fabricated blue TADF OLEDs and (**f**) lifetime (at the initial luminance of 500 cd/m^2^) of devices with or without PSVA.

Figure 2 shows the performance of the device.

**Figure 3:**
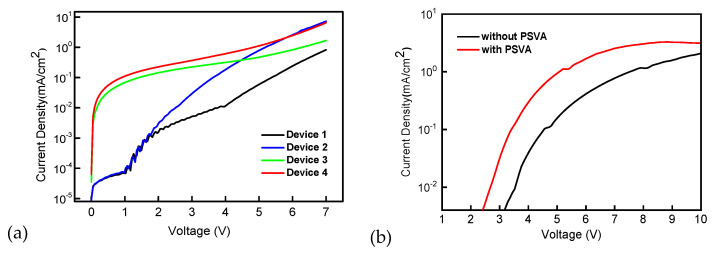
JV characteristics of hole-only devices (**a**) and electron-only devices (**b**).

The normalized EL spectra of devices 1–4 at the same brightness are shown in Figure 2a, and the changes in the EL spectra and CIE coordinates of devices 1 and 4 at different current densities are shown in Figure 2b,c. It can be seen that the spectra of these devices were stable. As shown by the J-V-L curves (Figure 2d), the current density and luminance of device 2 were higher than those of contrast device 1 at first, and then decreased. The increase in current density at low voltage led to the improvement of efficiency; the main factor was the increase in electron injection, which was evidenced by the higher electron current of the electron-only device, as shown in Figure 3b. When the PEDOT:PSS layer was processed by PSVA, as in device 3, the current density increased, but the luminance and efficiency, especially at low voltage, decreased significantly. This was because the conductivity of the PEDOT:PSS layer increased after PSVA treatment, and the injection barrier of the holes and work function reduced [17], resulting in an increase in hole current of device 3 at low voltage, which was confirmed by the corresponding current result shown in Figure 3a. Nevertheless, after the increase in holes, there were not enough electrons to match them to form excitons, leading to more quenching and lower current efficiency. The current density of device 4, in which both the PEDOT:PSS and EML treated by PSVA clearly decreased, and the highest brightness levels increased, the maximum luminance was 1638 cd/m^2^, which means that both carriers injected effectively and mostly balanced. It can be seen from the CE-L and EQE-L (Figure 2e) that the current efficiency of device 4 increased and the degree of efficiency roll-off reduced from 33.3% to 26.6%. The charge balance improved the device’s stability, reduced exciton quenching and improved the device’s lifetime (which was tested in air). Specific data results are listed in Table 1.

The single-carrier devices in which the PEDOT:PSS and EML were processed, corresponding to device 1 to 4, were prepared to confirm the above analysis. The hole-only devices were fabricated with the structure as:

ITO/PEDOT:PSS/mCP:DMAC-DPS/TCTA (25 nm)/MoO_3_ (30 nm)/LiF/Al

The electron-only devices were fabricated with the structure as:

ITO/ZnO/EML (without or with M)/DPEPO (10 nm)/TPBi (30 nm)/LiF/Al

It is clear that in the hole-only devices, as shown in Figure 3a, the current density of device 3 and device 4 started to increase when the voltage was less than 1 V, indicating that a small number of holes was injected through the PEDOT:PSS layer processed by PSVA to lower the work function, thus reducing the hole injection barrier, which is consistent with previous research results [17]. However, under low bias, the current form of device 2 was the same as that of device 1. These results mean that the injection of holes under low bias is dominated by the properties of PEDOT:PSS. Under high bias, the hole current of device 2 increased greatly compared with device 1, which was consistent with the difference between device 3 and device 4. This shows that the hole current is dominated by EML under high bias. If EML was processed by PSVA, the hole current increased obviously. In addition, the electron current, as shown in Figure 3b, also increased and the initial injection voltage appeared to decrease after EML treatment by PSVA. Therefore, it is concluded that PSVA processing can improve the mobility of holes and electrons in the EML and improve their injection at the same time.

**Figure 4:**
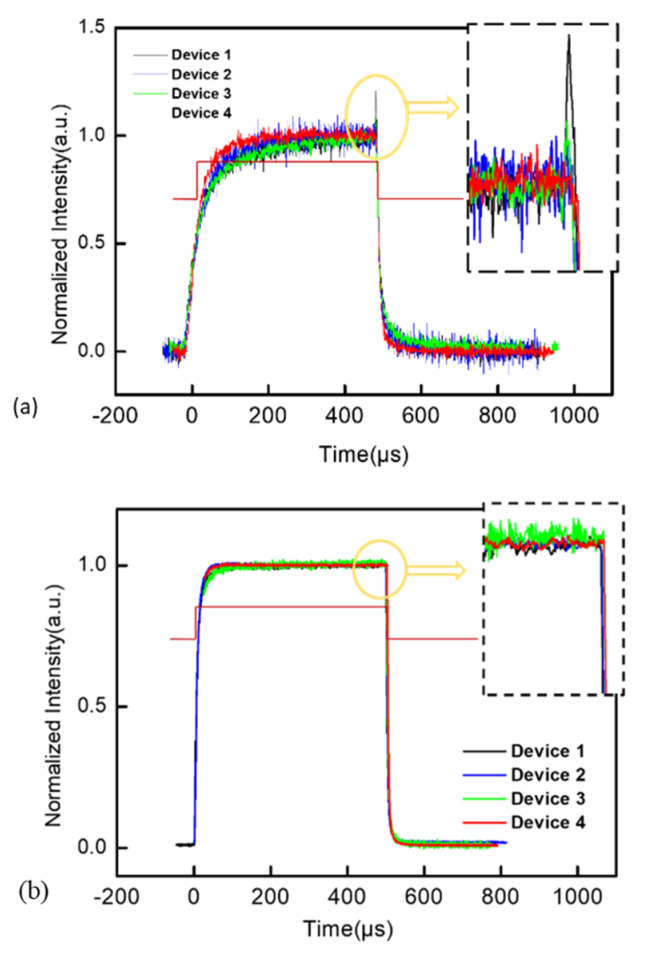
Transient EL characteristics of the device at low current density of 4 mA/cm^2^ (**a**) and high current density of 40 mA/cm^2^ (**b**).

The transient EL intensity of four devices at the same current density is shown in Figure 4. For the transient measurement, we set the pulse signal period to 1 ms and the forward bias duration to 500 us to ensure that the luminescence of all the devices would reach a steady state. The luminance of the devices at a low current of 4 mA/cm^2^, as shown in Figure 4a, are determined by the number of injected carriers. Traps in devices capture carriers until traps are filled. Carriers then combine to form excitons for radiative emission. If there are enough injected carriers and low-density traps, the number of carriers used to form excitons in EML for luminescence quickly reaches a steady state; therefore, the time taken for the luminescence to reach a steady state is greatly shortened. According to the response time of the rising edge, device 4 reached the luminescence steady state fastest, which means that device 4 had a lowest trap density among all the devices; consequently, more carriers could recombine more quickly for emission. Therefore, the amount of charges, stored by the traps inside device 4, that was required to form polarons was smaller, which resulted in a decreased probability of charge quenching and improved efficiency roll-off. The second-fastest time taken to reach the electroluminescence steady state, behind device 4, was that of device 2. This is consistent with the J-V-L curve. The EMLs in both device 2 and device 4 were treated with methanol vapor annealing. At low current density, electrons are minority carriers. PSVA improves the quality of EML, as well as improving the electron injection and transport. Therefore, electrons can be injected into EML more effectively and recombine with holes at low current density. When the forward bias was switched, a sudden change in the electric field would cause trapped charges inside the devices to be released, which was reflected in the TREL test as an overshoot intensity signal. Devices 1 and 3 without PSVA-treated EML showed a strong overshoot, which was due to the electrons accumulating at the interface flooding into the EML after the forward bias was switched. In particular, untreated device 1 had the largest overshoot due to the significant accumulation of electrons and holes at the interface. After the PEDOT:PSS was treated with PSVA in device 3, the accumulation of holes between the EML and PEDOT:PSS layer decreased; consequently, the overshot decreased in turn.

**Figure 5:**
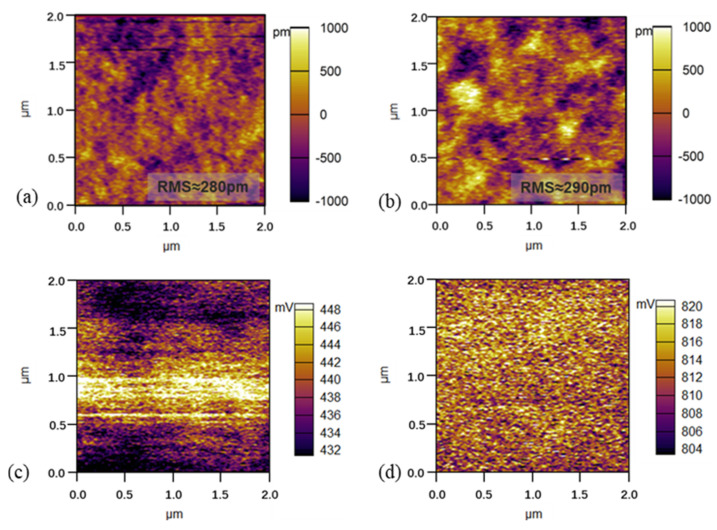
AFM images of (**a**) EML without PSVA, (**b**) EML with PSVA; SKPM images of (**c**) EML without PSVA, (**d**) EML with PSVA.

To explore the influence of PSVA on the film formation of organic small molecules, we used an atomic force microscope (AFM) and scanning Kelvin probe microscopy (SKPM) to test whether the EML annealed in methanol vapor (Figure 5). The results show that the roughness of the EML treated with methanol vapor was 290 pm, which was similar to that of the film without methanol vapor (280 pm). However, there is a large dark area in the upper right corner of Figure 5a, which indicates that the film formation was not uniform. The film distribution in Figure 5b is more dispersed and has better uniformity than the film in Figure 5a.

It is known that the transport of carriers in organic molecules is accomplished by carrier transition between different local states. Due to the uneven arrangement of molecules during the spin-coating process, many interfacial states and defects are formed in organic small molecule films. These local state traps, especially at the interface of two layers, have a strong ability to bind carrier injection [22]. Therefore, we measured the surface potential of EML to analyze the trap states on the EML surface. As seen from the test images of SKPM, the potential of the EML treated with methanol vapor increased significantly, from 439 mV to 812 mV. The film potential was affected by intermolecular anisotropy and changes in crystal defects, charge traps, and the film’s microstructure. An increase in EML surface potential can be determined by the reduction in the number of surface electron vacancies after PSVA treatment, leading to an upward shift in the Fermi energy level and a decrease in the EML surface work function. The disordering of organic molecules creates many defects at the interface and carriers are bound in a localized state. Electrons are injected through the surface of EML, which is strongly influenced by surface traps. For the whole OLED, the potential of EML uniformity increases, and the electron traps decrease, which reduces the charge capture of the device by the interface defect state when contacting DPEPO/ETL, and increases the charge utilization rate.

**Figure 6:**
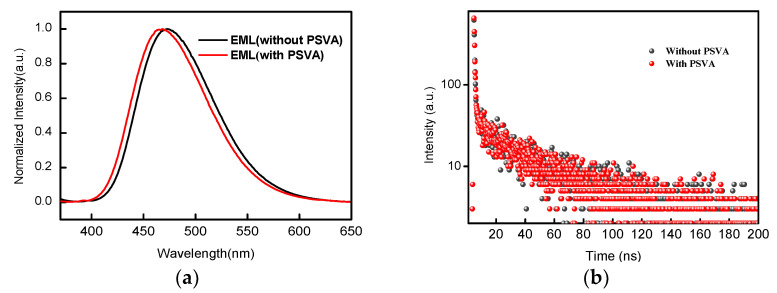
(**a**) PL spectra of mCP: 20 wt% DMAC-DPS films with or without PSVA; (**b**) transient PL decay of mCP: 20 wt% DMAC-DPS films at room temperature.

The photoluminescence (PL) spectrum of the DMAC-DPS-doped films with mCP as the host was tested (Figure 6). The test showed that the PL of the PSVA-treated EML was blue-shifted compared to the untreated EML film. However, the electroluminescence (EL) spectrum did not show a change in peak position (Figure 6a). It is well known that the excitation energy of electroluminescence is high and that the exciton energy level is complex. Molecular arrangements and interface defects have little effect on the peak position of EL. The excitation energy of PL comes from photons, and exciton energy is generally fixed in a simple mode. The single excitation type makes the luminescence spectrum sensitive to the phase transition of molecules. Figure 6b displays the transient PL decay curves at 470 nm for the DMAC-DPS doped films; both EML films showed the same prompt lifetime of 20 ns. This confirms our conjecture that no other functional groups would be introduced into the organic films after polar-solvent treatment.

**Figure 7:**
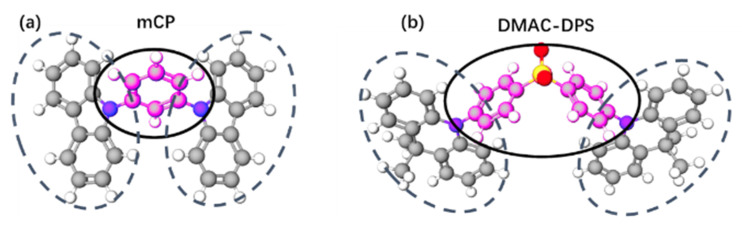
The molecular structures of (**a**) mCP and (**b**) DMAC-DPS. The part marked by the solid line represents the distribution of the molecular LUMO, the dotted line represents the distribution of the molecular HOMO.

The LUMO and HOMO level distribution of mCP and DMAC-DPS are shown in Figure 7. We simulated and calculated the polarity of these molecular groups and their solubility in polar solvents based on the algorithm provided by http://www.swissadme.ch/ (accessed on 19 October 2021). The calculation results are shown in Table 2. It was found that the receptor groups of these two molecules, that is, the molecular parts with LUMO energy level distribution, had stronger polarity and better solubility in polar solvents than the parts with HOMO energy level distribution. Methanol is a highly polar solvent. We speculate that during PSVA, the LUMO parts of molecules with higher polarity tend to be exposed to methanol vapor and gradually approaches the surface. Next, the HOMO part tends to move inward, away from methanol gas. In the structure of the device, this rearrangement of molecules is beneficial to the injection of electrons from the ETL to the interface of the EML. The orderly arrangement of molecules makes the charge transmission channels smoother and reduces the number of traps at the interface, which would lower the initial injection voltage and improve the mobility of electrons.

**Figure 8:**
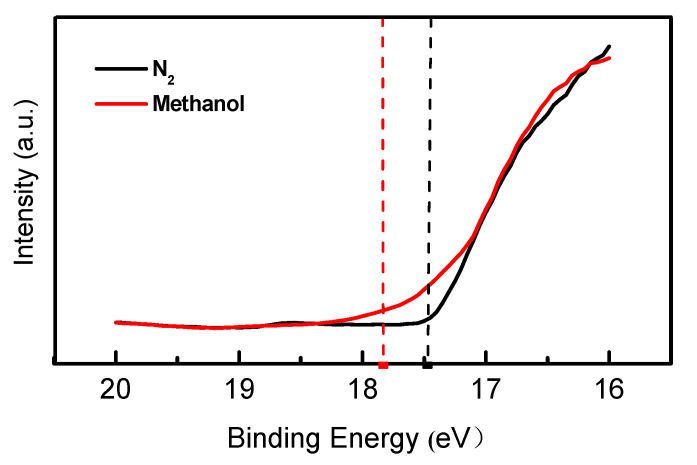
UPS spectra of EML with or without methanol vapor annealing.

Therefore, we tested UPS on the EML surface (shown in Figure 8) to confirm the above analysis. After PSVA, the surface work function of the EML decreased by about 0.36 eV, which was consistent with the SKPM result. The Fermi level of the EML surface moved upward as a result of PSVA treatment. After the electron trap filled, the empty electron state on the surface decreased, so the work function decreased. The decrease in the electrons accumulated at the interface under low current density on the transient EL test and the improvement of the electron injection on the J-V test of single-carrier devices support our conjecture that PSVA the post-processing method enhances the electron injection of EML surfaces.

Based on these results, it is concluded that PSVA treatment affects the path of molecular movement and the arrangement of organic molecules in the transformation of EML films from the liquid phase to solid phase. The LUMO parts in the molecules tend to move to the interface between the EML film and methanol vapor. On the one hand, after a part of the electron traps is filled, the empty electron state on the EML surface decreases, and it is easier to inject charge from DPEPO to the EML interface; on the other hand, the orderly arrangement of molecules results in the more effective transmission of the charges to the inside of the EML, increasing the transmission channels, thus improving the carrier balance, increasing the device performance and reducing the efficiency roll-off.

## 4. Conclusions

In summary, polar-solvent vapor annealing (PSVA) was used in both layers of solution-processed films, the PEDOT:PSS layer and the mCP:DMAC-DPS (EML) layer, in sky-blue OLEDs. The EL spectra with the peak at 470 nm corresponded to the emission of DMAC-DPS in all the OLEDs. The current efficiency was improved from 24.8 mA/cm^2^ to 26.7 mA/cm^2^, the highest brightness increased from 1246 cd/m^2^ to 1638 cd/m^2^, the efficiency roll-off ameliorated from 33.3% to 26.6%, and the device’s lifespan was also improved. An interesting finding is that the work function at the interface between EML and DPEPO was reduced from 3.73 eV to 3.37 eV, while the initial injection voltage of electrons decreased. According to our simulation of the solubility of the LUMO and HOMO molecular parts of the organic small molecules mCP and DMAC-DPS, the LUMO parts of these two molecules had stronger polarity than the HOMO parts, and had higher solubility in polar solution. By analyzing the data, we conclude that the LUMO parts of molecules tend to approach the surface and a more orderly molecular arrangement enhances carrier transport. After the PSVA treatment of EML, the surface morphology of the film becomes homogeneous and localized state traps are reduced, thus promoting electron injection. Through PSVA treatment of the PEDOT:PSS and EML layers, both hole injection and electron injection were improved respectively; furthermore, the carriers were more balanced, the quenching of the charges accumulated at the interface was reduced, and the current efficiency was increased.

## Figures and Tables

**Table 1:** Comparison of the device performance of solution-processed blue OLEDs, based on DMAC-DPS.

Device	EL_peak_ (nm)	V_on_ (V)	L_max_ (cd/m^2^)	CE_max_ (cd/A)	EQE_max_ (%)	Roll-off_400_^a^ (%)
1	470	4.2	1246	24.8	13.8	33.3
2	470	3.6	1398	27.0	15.2	30.9
3	470	4.5	1270	18.3	10.2	21.6
4	470	3.9	1638	26.7	15.0	26.6

^a^ The external quantum efficiency when luminance is 400 cd/m^2^.

**Table 2:** The solubility in polar solvents and lipophilicity of LUMO and HOMO molecular groups.

Material	Type	Solubility in Polar Solvent	Lipotropy
**mCP**	LUMO	2.96 × 10^−2^ mg/mL	3.39
	HOMO	3.52 × 10^−6^ mg/mL	8.07
**DMAC-DPS**	LUMO	1.49 × 10^−1^ mg/mL	2.4
	HOMO	7.83 × 10^−3^ mg/mL	4.34

## Data Availability

The data presented in this study are available on request from the corresponding author.
